# Advanced Merkel cell carcinoma in setting of pembrolizumab therapy for squamous cell carcinoma

**DOI:** 10.1016/j.jdcr.2024.03.003

**Published:** 2024-03-11

**Authors:** Henry Jeon, Keith Mackenzie

**Affiliations:** a Midwestern University Arizona College of Osteopathic Medicine, Glendale, Arizona; b Mackenzie Dermatology, Prescott, Arizona

**Keywords:** chemotherapy, hyperprogressive disease, immunotherapy, medical dermatology, Merkel cell, Merkel cell carcinoma, metastatic, nonmelanoma skin cancer, paradoxical reaction, PD-1, pembrolizumab, skin cancer, squamous cell carcinoma

## Introduction

Merkel cell carcinoma (MCC) is a neuroendocrine tumor of unclear cell origin characterized by high recurrence, early metastasis, rapid growth, and poor prognosis.^1^ Patients of old age, immunosuppression, and extensive UV exposure are at increased risk of the malignancy.^2^ Tripling its incidence in the past 15 years, MCC has been of increasing dermatological concern and is currently managed via sentinel lymph node biopsy with definitive excision, followed by radiation therapy or immunotherapy.^1^, ^2^, ^3^

Initially implemented for treatment of refractory melanoma, pembrolizumab is food and drug administration approved for the treatment of squamous cell carcinoma (SCC), MCC, and numerous advanced cancers.^4^ Pembrolizumab is a programmed cell death receptor 1 (PD-1) inhibiting IgG4 monoclonal antibody that prevents PD-1 induced T-cell inactivation by tumor cells.^5^ Adverse reactions include skin reactions such as Stevens-Johnson syndrome and bullous pemphigoid, endocrinopathies, hepatotoxicity, nephrotoxicity, and allergic reactions.^4^

Among patients receiving immunotherapy, a rare phenomenon termed hyperprogressive disease (HPD) has been found to occur.^6^ First documented in 2016, this poorly understood mechanism manifests as a malignancy paradoxically experiencing accelerated growth after immunotherapy initiation.^7^ Numerous case reports and studies explore HPD but fail to address application of this concept in different disease models such as newly arising tumors or cancer phenotype alterations.^8^

We report a case of paradoxical MCC occurring in the setting of pembrolizumab therapy for advanced SCC.

## Case report

We report an 86-year-old female patient receiving pembrolizumab treatment for advanced SCC who presented with a painful, nonbleeding, nonpruritic, raised lesion on her head. The patient initially noticed her lesion 2 weeks prior to presentation, 2 weeks into her first immunotherapy session, and reported rapid growth of the mass.

Pembrolizumab was originally initiated by oncology to treat multiple poorly controlled, progressive SCCs of the head and neck. On initial presentation, the patient had 8 SCCs of the head and neck region ranging from SCC in situ to moderate-to-poorly differentiated. The patient has a history of over 50 cutaneous SCCs, with 20 occurring within the past 2 years. Past SCCs were managed with local excision, 5-fluorouracil, and radiation therapy. The patient had an additional apast medical history of essential thrombocythemia, chronic kidney disease, deep vein thrombosis, atrial fibrillation, hypertension, and leukemia. Concomitant medications included amlodipine, metoprolol, triamterene-hydrochlorothiazide, warfarin, anagrelide, ruxolitinib, and pembrolizumab. In addition, she had an extensive history of tanning bed use.

**Fig 1:**
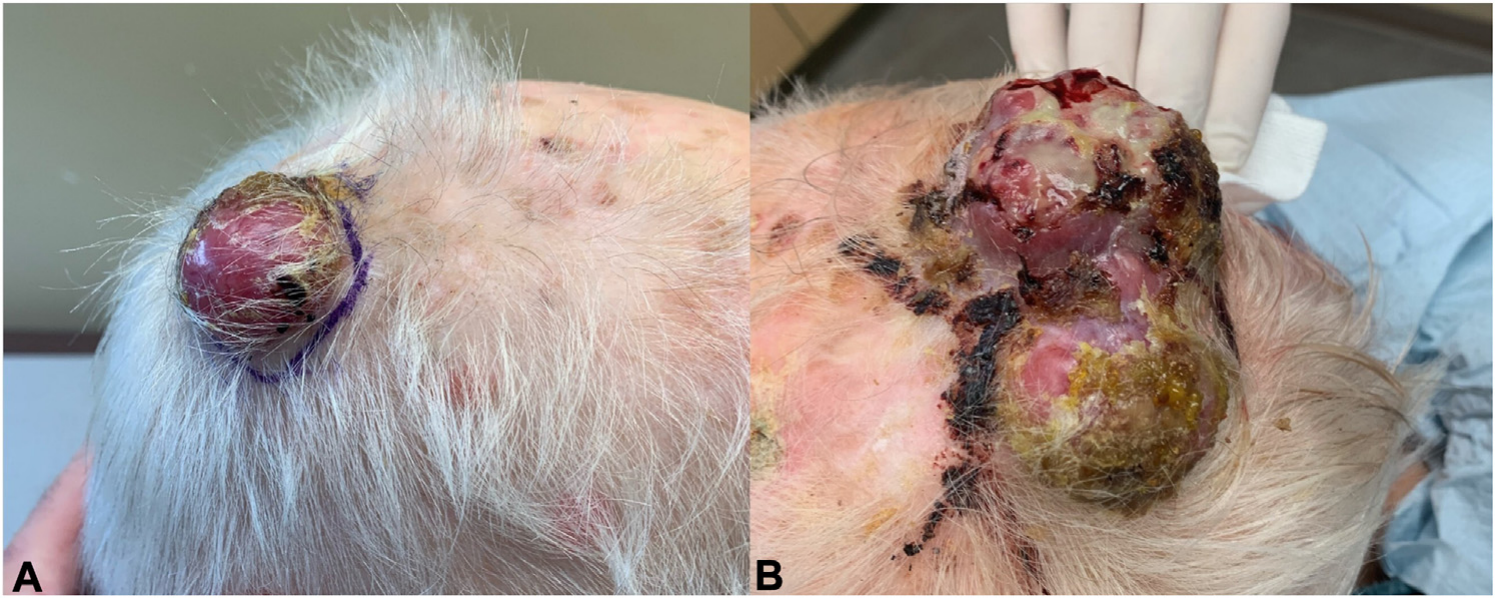
Merkel cell carcinoma of scalp. **A,** Solitary lesion at initial presentation. **B,** Two new lesions on 5-week follow-up.

Local examination revealed a firm nonmobile 3.5 × 3.5 × 2.0 cm erythematous papule with associated heme crusting and secondary impetiginization on the right posterior lateral vertex of the scalp (Fig 1, *A*). A partial resection of the mass was performed due to high clinical suspicion of MCC and was sent for histopathology and with referral to oncology for a positron emission tomography scan.

**Fig 2:**
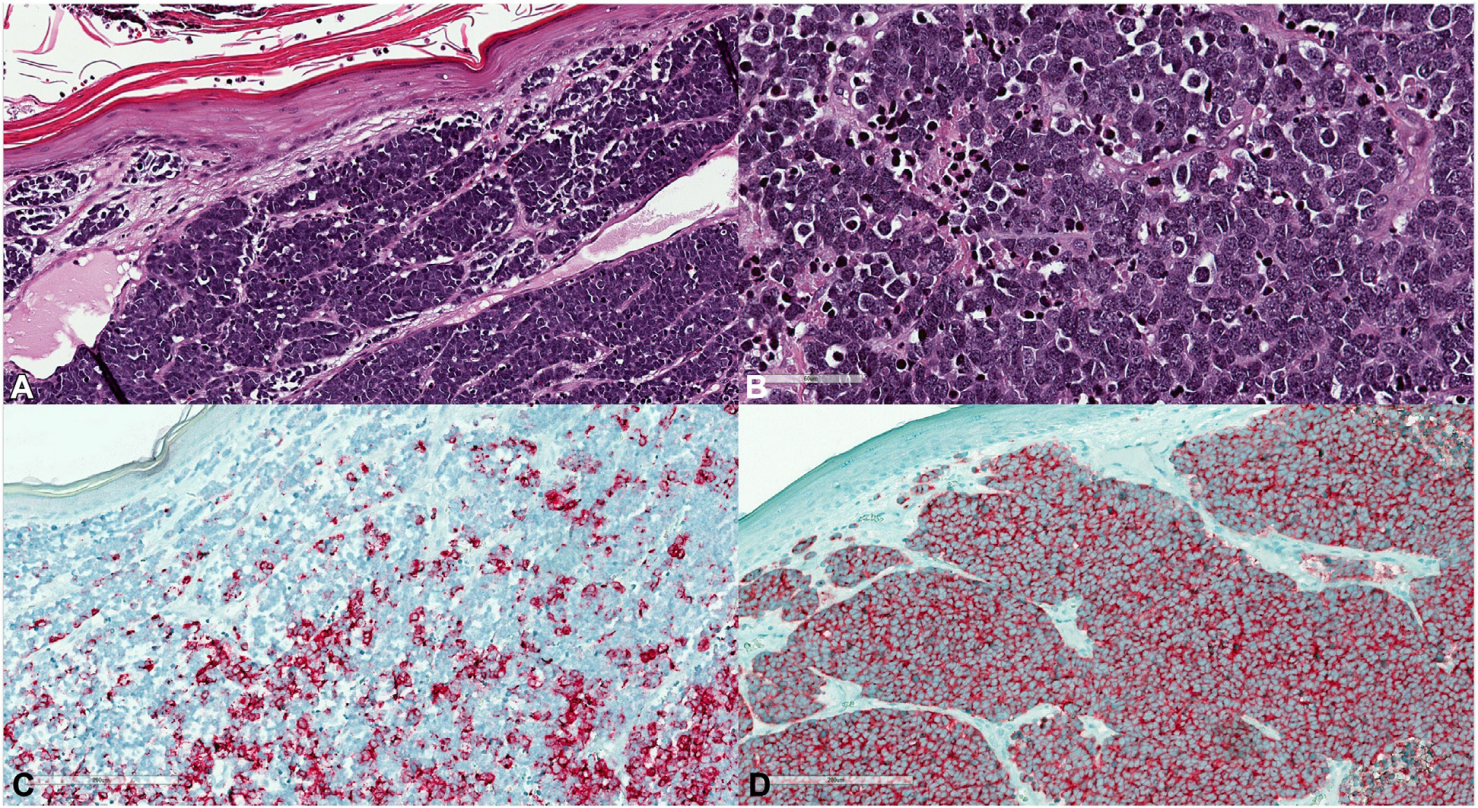
Histopathology of the Merkel cell carcinoma. **A,** Sheet-like to trabecular proliferation of relatively uniform, small, round to oval cells (Hematoxylin-eosin stain; original magnification × 10). **B,** Neoplastic cells will finely dispersed chromatin, numerous mitotic figures, and single-cell necrosis (Hematoxylin-eosin stain; original magnification × 40). **C,** Punctate paranuclear pattern positive for cytokeratin (Original magnification × 10). **D,** Immunohistochemistry with synaptophysin (Original magnification × 10).

Dermatopathology report revealed findings consistent with primary cutaneous neuroendocrine carcinoma, also known as MCC, extending to base and edge with lymph vascular and fatty invasion, classified as stage pT2. Microscopic description included sheet-like to trabecular proliferation of relatively uniform, small, round to oval cells with finely dispersed chromatin, numerous mitotic figures, and single-cell necrosis (Fig 2, *A* and *B*). The tumor cells had scant cytoplasm that is reactive in a punctate paranuclear pattern positive for cytokeratin, synaptophysin, and chromogranin, but negative for tumor protein p63 (Fig 2, *C* and *D*).

Positron emission tomography scan findings revealed an intensely hypermetabolic exophytic lesion of the right vertex scalp, compatible with MCC, along with bilateral mild to moderately hypermetabolic cervical chain lymph nodes, highly suspicious for regional nodal metastases.

Upon follow-up 5 weeks later, the patient presented with 2 new similar but larger lesions around the same area of her scalp, the first measuring 8.0 × 6.0 × 4.0 cm on the right posterior vertex and the second measuring 5.0 × 4.0 × 3.0 cm on the right posterior occipital scalp (Fig 1, *B*).

**Fig 3:**
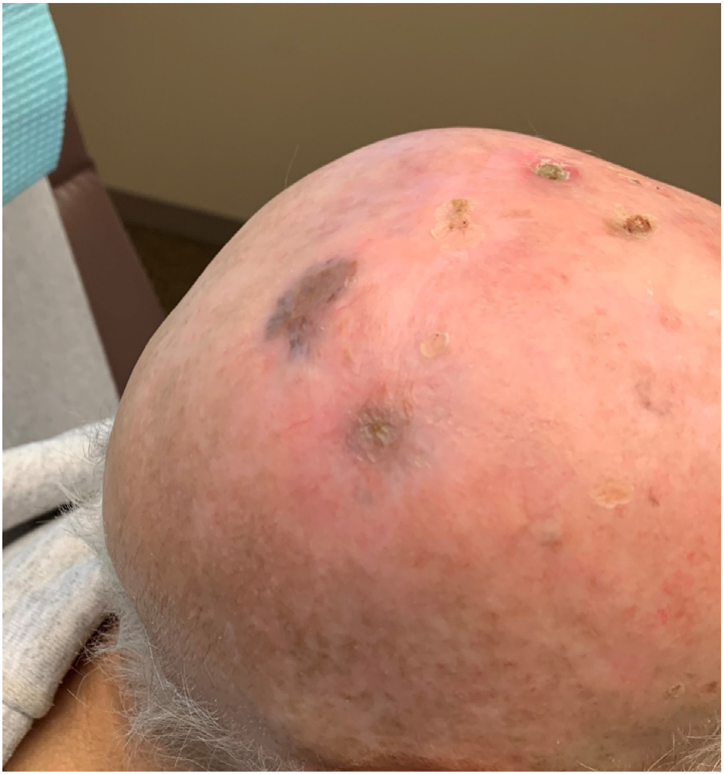
Merkel cell carcinoma of scalp postradiation therapy on 4-month follow-up.

Immunotherapy was stopped, and surgical intervention options were discussed with the patient. The patient declined surgery and palliative radiation therapy was initiated. Upon follow-up 4 months later, the patient’s MCCs exhibited a significant decrease in size with a remaining nonraised area of postinflammatory hyperpigmentation, and pembrolizumab therapy was restarted at this time (Fig 3). Repeat positron emission tomography scan revealed no lymph node involvement and no evidence of active disease.

## Discussion

The occurrence of MCC in a patient receiving pembrolizumab treatment presents a paradoxical scenario due to the immunotherapy being approved to treat the condition itself. With her advanced age, history of multiple malignancies, and personal history of UV exposure, the patient is already at a high risk for developing MCC. However, whether the cancer was developing prior to onset of immunotherapy and inadvertently but poorly controlled by it, or an HPD was manifesting, is unclear.

The pathophysiology of HPD is explained by either of 2 leading theories—adaptive immunity to immunotherapy or modified innate immunity. The first describes tumor cells’ ability to evade PD-1 inhibition via upregulation of other T-cell inhibition mechanisms. Notably, the upregulation of T-cell immunoglobulin mucin-3, an alternative immune checkpoint, has been observed in successful adaptive resistance and increased survival of tumor cells. Furthermore, T-cell immunoglobulin mucin-3 blockade in mice has demonstrated clinical benefit.^9^ PD-1 inhibition, beyond its effects on T cells, alters innate immune system functioning. Major findings reveal that PD-1 blockade can impair the ability for natural killer cells to produce perforins and granzymes, promote interleukin 10 release from type 3 innate lymphoid cells, dendritic cells, and monocytes, and hinder antigen presentation, overall promoting a pro-oncotic environment.^6^^,^^10^ These mechanisms may be applicable to our patient’s scenario, where pembrolizumab therapy may have induced HPD in a preexisting MCC or fostered development of a new MCC.

## Conclusion

This is a case of MCC occurring in the setting of pembrolizumab therapy for advanced SCC. HPD, a poorly understood phenomenon that occurs in patients receiving immunotherapy has only been documented to occur while being treated for a preexisting malignancy. The aggressive nature, rapid recurrence, and disease presentation of this patient’s MCC allow us to attribute the rare, paradoxical presentation to a potential HPD model applied to a de novo malignancy.

## Conflicts of interest

None disclosed.
